# Telesurgery and Robotics: An Improved and Efficient Era

**DOI:** 10.7759/cureus.14124

**Published:** 2021-03-26

**Authors:** Anmol Mohan, Um Ul Wara, Muhammad Taha Arshad Shaikh, Rahil M Rahman, Zain Ali Zaidi

**Affiliations:** 1 Medicine, Karachi Medical and Dental College, Karachi, PAK; 2 Medicine and Surgery, Karachi Medical and Dental College, Karachi, PAK; 3 Surgery, Karachi Medical and Dental College, Karachi, PAK; 4 Surgery, Abbassi Shaheed Hospital, Karachi, PAK; 5 Internal Medicine, Jinnah Medical and Dental College, Karachi, PAK

**Keywords:** telesurgery, telerobotic surgery, iot, 5g network, haptic feedback, tactile robotics

## Abstract

Telesurgery, or remote surgery, is widely known as a master-slave technology. It has achieved a milestone in surgical technology and intervention, providing widespread prospects of operating on a patient in a remote area with increased accuracy and precision. It consists of one or more arms controlled by a surgeon and a master controller in a remote area accessing all the information being transferred via a telecommunication system. This paper reviews the present advancements and their benefits and limitations in the field of telesurgery. A handful of operations have been done so far. However, due to time-lag (latency), global networking problems, legal issues and skepticism, and on top of the cost of robotic systems and their affordability have led to the concept of telerobotics and surgery to lag. However, with more information and high speed, 5G networking, which has been in a trial to reduce latency to its minimum, is beneficial. Haptic feedback technology in telesurgery and robotics is another achievement that can be improved; further, this allows the robotic arms to mimic the natural hand movements of the surgeon in the control center so that the master controller can perform surgeries with more dexterity and acuity. Due to coronavirus (COVID-19), this type of surgery approach can reduce the probability of contracting the virus, saving more lives and the future.

## Introduction and background

The concept of distance between a surgeon and a patient is not a new idea. It was explored in the 1970s by the U.S. National Aeronautics and Space Administration (NASA), which had a particular interest in treating the astronauts in space, based on the concept of remote surgery or telesurgery (TS) [1]. Simultaneously, the advent of robotic surgery (RS) made it possible for a surgeon to operate with increased dexterity, improved accuracy, and broader accessibility to diﬃcult locations in the body, such as the pelvis [2]. The significant advancements in telecommunications and robotic surgery throughout the 1980s and 1990s led to telerobotic surgery being considered a practical option.

The first telerobotic surgery was successfully performed on a patient in Strasbourg, France, by Professor Jacques Marescaux on September 7, 2001 which was called the 'Lindbergh Operation'. It was a telesurgery-assisted cholecystectomy, which was completed in 54 minutes without any complications [3]. It was a richly symbolic milestone in surgery, that led the foundations of globalization of surgical procedures.

Since this first telerobotic surgery in 2001, the development of remote surgery has been diﬃcult because of significant limitations of the network system. We are currently living through the fourth industrial revolution in all the fields of our lives and surgery is no exception. The future of surgery is transforming with the integration of new technologies such as the fifth-generation (5G) internet, artificial intelligence, haptic feedback technology, 3-dimensional (3D) printing, and nanotechnology [4].

In 2019, telerobotic spinal surgeries based on 5G network were performed on 12 patients in six hospitals from six diﬀerent cities in China. Sixty-two pedicle screws were implanted out of which 59 screws were grade A in Gertzbein-Robbins criteria, and three were grade B. A new pattern of "one-to-many" remote surgery was also explored with these clinical series, where the surgeon in the master control room was providing surgical care to the patients who were physically isolated at once. These 12 cases showed it is possible to provide minimal latency, high bandwidth, and reliable communication for medical services with no telecommunication error or network delay [5].

## Review

Benefits of telesurgery

There are many advantages of telesurgery as compared to conventional surgical methods. The advancements in telecommunication and robotic surgery (RS) have made telesurgery a promising and feasible option for patients to get treated without traveling much [6]. Some of the benefits of telesurgery are discussed below:

Eliminating Long Distance Travel

Traveling for medical care is not a feasible option for many either because of financial constraints, travel-related health risks, travel restrictions, or time delay, which in some cases can be counterproductive. Telesurgery is an excellent solution to get medical attention without patients' need to travel beyond their local hospitals through which surgical expertise can provide surgical care for patients from anywhere around the world [7].

Providing Healthcare to Medically Underserved Areas

Telesurgery promises to provide surgical care to people worldwide, especially the remote areas such as rural areas or battlefield and inaccessible areas such as spacecraft [7-10].

Surgical Collaborations

Collaborations between diﬀerent surgical experts from various healthcare centers can happen in real-time. A patient can benefit from the expertise of more than one healthcare specialist simultaneously. The complex microsurgical procedures can be benefitted from these real-time surgical collaborations [7,10].

Improving Surgical Accuracy

High definition (3D) cameras allow a surgeon to see the close-up views of the surgery site, not easily accessible [11]. Robotic arms make it possible to access the areas in the body which are very hard to access, such as the pelvis that is why urologists receive it well, colorectal and abdominal surgeons particularly [7,8,12]. Accelerometer technology is useful to cancel out a surgeon's physiological tremor and help the surgeons' anxiety, resulting in improved surgical accuracy and overcomes the limitations associated with surgeons' dexterity. Improved surgical accuracy reduces the chances of damaging surrounding structures, remarkably lessens the risks of blood loss, transfusions, and less risk of infection. In short, a patient can have a faster recovery and fewer complications (as in the case of the da Vinci Surgical System) [7,13].

Eliminate Potential Shortage of Surgeons

There is a global shortage of qualified surgeons and anesthetists [14] for which telesurgery can be a potential solution [7].

Minimizing the Risk of Infection

The coronavirus disease (COVID-19) caused by Sars-CoV-2 was declared a global pandemic on March 11, 2020 by the World Health Organization (WHO) [15]. During this time, surgical intervention is being reserved for only critical patients due to the risk of viral transmission. Telesurgery is a viable option for the protection of both the surgeon and the patient.

Limitations of telesurgery

Despite the promising advancements in surgery and telecommunications, some limitations still exist such as:

Global Network Development

To make telesurgery a feasible option for anyone in the world, there is a need to develop a good global network that allows patients in any part of the world to get treated without traveling for a long distance [7].

Legal Issues

The concept of getting medical care from a surgeon with no real interaction, can generate a degree of skepticism. The advent of telesurgery, on one hand has led to political and geographical limitations to be overcome, while on the other hand, it has given rise to several legal and ethical issues, as laws differ across the state and country borders [16].

Billing Issues

As different medical centers collaborate in telesurgery. The billing issue on distributing the participating medical centers' operation fees can be a barrier [7].

Equipment Acquisition

Acquisition and maintenance of the equipment is another hurdle [7].

Cost

The cost of robotic systems and the aﬀordability of high-speed telecommunication is a problem, especially in developing countries. The new robotic systems' costs are likely to be cheaper as more companies will get a license for manufacturing them [8].

Cyber Security Threats

The rapid growth in telesurgery applications has raised cyber-attacks against teleoperated surgical robots. These robotic computer systems have a possibility of being taken over and even being turned into a weapon which can be potentially harmful [17].

Latency

Time-lag is the major drawback observed in the field of telerobotic surgery. It refers to the delay in transferring sensory and motor modalities between two far-reaching locations [6]. Increased latency can increase the chances of inaccuracies, along with a lengthy operation [18]. Latency up to 200ms is known to be acceptable however, Korte et al. concluded that the time delay of more than 2 seconds results in unacceptable inaccuracies and ineﬃciency even when the most experienced teleoperator is performing, further highlighting the importance of this variable in telesurgery [18].

Proposed approach

Our proposed approach is the integration of all the emerging technologies that can result in a tremendous improvement in telesurgery.

High Speed 5G Network

The theoretical maximum speed of 5G is up to 10 Gbps, which means that it is a hundred times faster than 4G whose theoretical maximum speed is 100 Mbps [19]. High speed 5G internet incorporation with telesurgery will reduce the current latency period of 0.27 to 0.01 seconds [20]. This improvement in latency can solve the time delay problems that are an issue related to telesurgery. In addition, a fifth-generation (5G) network also allows a haptic application to take life [21]. All these improvements mean that it is a crucial technology for remote surgery.

Haptic Feedback and Tactile Robotics

In a telesurgery operating room, there is a high-resolution 3D - 360-degree view-camera and a robotic system is required. The surgeon looks at the patient's surgery site on a screen and uses his haptic arm to place the robotic arm in the operation theatre in a proper way. The tactile robot system interprets the surgeon's hand movements on the console precisely into the corresponding movements of the robotic arms. This makes it possible for robotic arms to mimic the surgeon's natural hand movements in the control center, allowing surgical experts to have more control over the instruments and increased dexterity than in a conventional operation room with conventional surgical instruments. Haptic technology allows the transmission of tactile information to reach the teleoperator [22]. Because of the invention of artificial intelligence, a new augmented and virtual reality surgery training program can use these technologies to improve the robotic arm [13] further and make it more precise with the integration of advanced tactile sensors [23].

One-to-Many Remote Surgery

A new concept of 'one-to-many' remote surgery is based on a surgeon simultaneously performing surgeries on multiple patients. It is a way through which surgical expertise can reach more patients. Previously, the concept of one-to-many remote surgeries had a lot of restrictions and limitations regarding time-lag. Thanks to the high-speed 5G internet, which has reduced the latency and has increased bandwidth, time-lag is no longer a limitation. The concept of 'one-to-many' remote surgery was successful in the 2019 telerobotic surgical series [5].

Internet of Things (IoT)

IoT is the new technological revolution that aspires to connect all the everyday physical objects to the internet, making a substantial global network of unique things that can share information amongst each other and complete scheduled tasks [24]. It is a revolutionizing technology that makes it possible to visualize instruments usage in specific procedures. The regular use of IoT in surgery will make it possible for us to visualize surgical procedures. It will bring multiple advances in medicine, such as in surgery [25].

## Conclusions

Telesurgery or remote surgery is a promising surgical advancement, however faces many challenges. Zero-latency time and improvement in haptic feedback technology are required for precise and well-done surgeries. Technologies like 5G network, IoT, and tactile robotics should be integrated in telesurgery to overcome these barriers. Cost and legalization to address legal and ethical issues remain to be addressed. Robotic surgery can demonstrate a pivotal role in the surgical procedures being performed in the current pandemic by minimizing the number of surgical staff in the operation theaters, hence curtailing the risk of COVID-19 infection that can subsequently lead to profound morbidity and mortality.
